# Caffeine Ingestion after Rapid Weight Loss in Judo Athletes Reduces Perceived Effort and Increases Plasma Lactate Concentration without Improving Performance

**DOI:** 10.3390/nu6072931

**Published:** 2014-07-22

**Authors:** Joao P. Lopes-Silva, Leandro J. C. Felippe, Marcos D. Silva-Cavalcante, Romulo Bertuzzi, Adriano E. Lima-Silva

**Affiliations:** 1 Sport Science Research Group, Department of Physical Education and Sports Science (CAV), Federal University of Pernambuco (Universidade Federal de Pernambuco), Alto do Reservatório street, Bela Vista, Vitória de Santo Antão, Pernambuco 55608-680, Brazil; E-Mails: joao_judo@hotmail.com (J.P.L.-S); leandrocamati@hotmail.com (L.J.C.F.); cavalcantemds@hotmail.com (M.D.S.-C.); bertuzzi@usp.br (R.B.); 2 Endurance Sports Research Group, School of Physical Education and Sport, University of São Paulo, Mello Moraes Avenue, Butantã, São Paulo, São Paulo 05371-140, Brazil

**Keywords:** rapid-weight loss, judo, caffeine, perceived effort, plasma lactate

## Abstract

The objective of this study was to examine the effect of caffeine on judo performance, perceived exertion, and plasma lactate response when ingested during recovery from a 5-day weight loss period. Six judokas performed two cycles of a 5-day rapid weight loss procedure to reduce their body weight by ~5%. After weigh-in, subjects re-fed and rehydrated over a 4-h recovery period. In the third hour of this “loading period”, subjects ingested a capsule containing either caffeine (6 mg·kg^−1^) or placebo. One hour later, participants performed three bouts of a judo fitness test with 5-min recovery periods. Perceived exertion and plasma lactate were measured before and immediately after each test bout. Body weight was reduced in both caffeine and placebo conditions after the weight loss period (−3.9% ± 1.6% and −4.0% ± 2.3% from control, respectively, *p* < 0.05). At three hours after weigh-in, body weight had increased with both treatments but remained below the control (−3.0% ± 1.3% and −2.7% ± 2.2%). There were no significant differences in the number of throws between the control, caffeine or placebo groups. However, plasma lactate was systemically higher and perceived exertion lower in the subjects who ingested caffeine compared to either the control or placebo subjects (*p* < 0.05). In conclusion, caffeine did not improve performance during the judo fitness test after a 5-day weight loss period, but reduced perceived exertion and increased plasma lactate.

## 1. Introduction

In judo competition, division by weight-class guarantees matched strength, agility, and power between the competitors [1,2]. However, athletes frequently reduce their body weight immediately before competition to reach the maximum weight for a lighter division, thereby obtaining advantages against lighter opponents [2,3]. This rapid weight loss typically starts 3–5 days before competition, when athletes may restrict food and fluid, exercise in rubber or plastic suits, vomit, and use saunas or diet pills [4,5]. However, these weight-loss procedures can lead to hormonal imbalance [4], hydroelectrolytic imbalance [5], hyperthermia [6], cardiovascular distress [7], impaired immune function [8], and moodiness [9]. Importantly, these physiological alterations can reduce anaerobic capacity, an important determinant of overall performance in judo [10,11].

It is important to note that most of previous studies investigating the effects of rapid weight-loss on performance did not allow athletes to re-feed and rehydrate after the weigh-in [10,11,12,13]. This strategy differs from common practice in actual judo competitions, in which a short period (~4 h) after the weigh-in is allowed for recovery. Therefore, this “loading period” between weigh-in and competition provides an opportunity to develop strategies that minimize the negative effects of rapid weight loss and optimize performance. Additionally, studies investigating the effects of weight loss on performance utilized laboratory-based techniques, which may not reflect the demands of real judo combat. Therefore, a judo-specific performance test (Special Judo Fitness Test, SJFT), which is more representative of judo movements than laboratory tests, has been proposed as a valid and reliable measure of performance in judo athletes [14,15,16].

Caffeine is a potentially useful ergogenic resource for the short loading period after weigh-in. Caffeine ingestion (3–9 mg·kg^−1^) significantly improves performance during short-term, high-intensity exercises [17,18], sprints [19,20], and strength and power exercises [21]. Caffeine may also promote anaerobic glycolysis, increasing anaerobic capacity [22]. Additionally, caffeine affects the central nervous system (CNS) by acting as a potent adenosine receptor antagonist [23]. It easily crosses the blood-barrier by both simple diffusion and carrier-mediated transport, and competes with adenosine for binding sites on the adenosine A2 receptor [24]. Adenosine is molecularly similar to caffeine, and enhances pain perception and induces sleepiness. Caffeine can block these negative effects and reduce the rating of perceived exertion (RPE) [25,26], thus improving performance. Caffeine is therefore a strong candidate to improve judo performance, but its effect on performance after a rapid weight-loss period has not been tested.

The objective of this study was to examine the effect of caffeine on judo performance following a 5-day weight loss period and a 4-h loading period. Plasma lactate and RPE responses were measured to provide insight into anaerobic metabolism and perceived effort. We hypothesized that caffeine ingestion would increase plasma lactate and reduce RPE, consequently improving judo performance.

## 2. Methods

### 2.1. Participants

Six experienced (weight category <60 kg (*n* = 2), <66 kg (*n* = 2), <73 (*n* = 1), and <90 kg (*n* = 1), four black belts and two brown belts) athletes participated in this study. All athletes were male (age 25.3 ± 5.7 years; weight 71.1 ± 13.5 kg; height 167.0 ± 3.6 cm; and body fat 13.2% ± 11.2%) and actively competing at the regional or national level with 14.4 ± 8.9 years of judo experience. At the time of data collection, athletes were 5.5% ± 3.4% above their weight class. The athletes were recruited from the local judo federation and all had experience with rapid weight loss procedures before the initiation of this study.

The required sample size was estimated from the equation *n* = 8 *e*^2^/*d*^2^, as suggested by Hopkins [27], where *n*, *e* and *d* denote sample size, coefficient of variation, and magnitude of the treatment effect, respectively. Coefficient of variation was assumed to be 0.73 [28]. Anticipating a treatment effect with a magnitude of 3.1% [29], detection of a conservative, statistically significant 1% difference would require at least five participants. To account for athlete attrition, we aimed to recruit eight participants. However, two participants failed to reduce their body weight during the experimental period. Thus, statistical analysis was performed on the six successful participants. All experimental sessions were scheduled during a period with no official competitions. The experimental protocol was approved by the Ethics and Research Committee of Maceio University Center (CESMAC, protocol number: 1417/12). This study was conducted in accordance with the International Ethical Guidelines and Declaration of Helsinki.

### 2.2. Experimental Design

**Figure 1:**
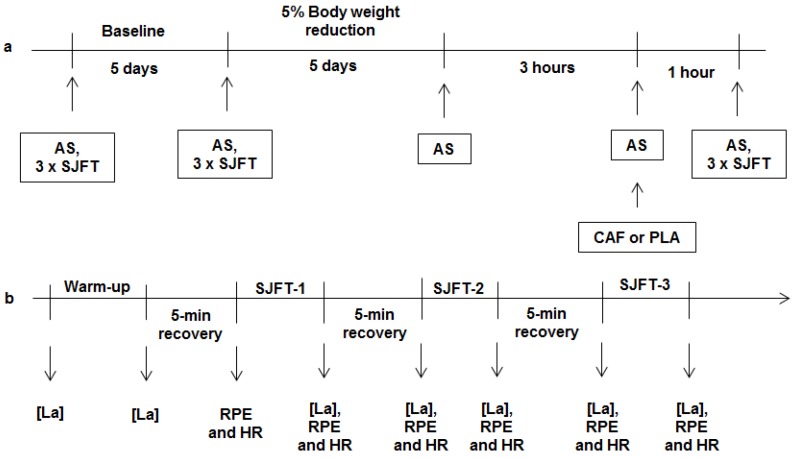
(**a**) Experimental design. (**b**) Performance protocol. AS: Anthropometric assessment; SJFT: Special judo fitness test; CAF: caffeine; PLA: placebo; [La]: plasma lactate concentration; RPE: rating of perceived exertion; HR: heart rate.

The experimental design is illustrated in Figure 1. Body weight, body fat, and performance during SJFT were assessed 3-h postprandially on the first and last day of a 5-day control period. Participants were asked not to exercise the day before these control tests. These data were used to calculate test-retest reliability and were averaged for post hoc comparison with caffeine and placebo. Athletes followed their normal diet (1664.4 ± 328.8 kcal; 227.5 ± 37.5 g/day of carbohydrate; 43.2 ± 16.1 g/day of fat; and 87.6 ± 28.9 g/day of protein) and training routines during this period. Athletes then performed two cycles of a 5-day, rapid weight loss period to reduce their body weight as they would in an actual competition. The athletes were then weighed (weigh-in) and body fat was measured before a 4-h recovery regimen (loading period). In the third hour of this loading period, body weight and fat percentage were determined and athletes ingested a capsule containing either caffeine or placebo. One hour later, participants performed three SJFT bouts with 5-min recovery periods. A 15-day, wash-out period was then applied between cycles. During the wash-out, the athletes were free to return to their normal training and food routine.

All tests were conducted at the same time of day to minimize circadian variance. The experimental sequence (caffeine or placebo) was performed in a double-blinded, counterbalanced and crossover manner. The athletes were instructed not to ingest alcohol during the study and to refrain from consuming caffeine-containing substances (*i.e.*, coffee, chocolate, and soft drinks) for 24 h before the experimental tests.

### 2.3. Body Composition

Body composition was estimated from three skinfold thicknesses on the right side (chest, abdomen and thigh). Body density was obtained as previously described by Jackson and Pollock [30], and body fat (%) was calculated using Siri’s equation [31]. Body weight was measured using a digital scale with 0.1 kg accuracy. Height was assessed using a stadiometer with scale of 0.1 cm.

### 2.4. Rapid Body Weight-Loss Procedures

Athletes were asked to lose ~5% of their body weight, using the same methods they would use if they were making weight for a competition [11,32,33]. Athletes were instructed to record these procedures and to repeat them in the second experimental session. Use of laxatives and diuretics to accelerate weight loss were prohibited, but athletes were free to manipulate food and fluid intake and exercise. Participants were free to consume food and water during the first three hours of the loading period after the weigh-in. All food and fluid consumed during this period was recorded and reproduced in the subsequent experimental session. The athletes were prohibited from consuming any food or water during the last hour of recovery.

### 2.5. Caffeine and Placebo Ingestion

Athletes ingested one capsule containing either 6 mg·kg^−1^ (control body weight) of pure caffeine (FHN pharmacy, Maceio, Alagoas, Brazil) or cellulose (placebo). At the end of each experimental condition, the athletes were asked to identify if they were able to distinguish which capsule they had ingested.

### 2.6. Judo Performance

Participants performed a warm-up consisting of three bouts of the tsuri-komi-gochi technique applied on a partner (10, 20 and 30 s for first, second and third bouts, respectively), separated by a 10-s recovery. The athletes then rested for 5-min, before performing three bouts of SJFT with 5-min recovery periods. The SJFT protocol is considered a reliable and reproducible means to measure judo performance [14,15]. Briefly, three athletes of similar body weight are needed to perform the SJFT: (1) one participant called TORI, who is the evaluated athlete and; (2) two participants called UKE, who will receive the throws. The TORI begins the test between the two UKEs, 3 m from one another. After a sound signal, the TORI runs to one UKE and applies a throwing technique “ippon seoi nague”. Then, TORI immediately runs to the another UKE and applies a new throw. The evaluated athlete (TORI) must complete as many throws as possible. Each SJFT bout was composed of three series (1 × 15 s, and 2 × 30 s) separated by 10 s for recovery. Performance was determined by the total throws completed during the SJFT. In addition, the following index was calculated [15,16]:

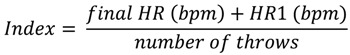
(1)
where final HR was the heart rate measured immediately after a given bout, and HR1 was the heart rate measured 1 min after this same bout.

### 2.7. Rating of Perceived Exertion, Plasma Lactate Concentration and Heart Rate

RPE was measured before and immediately after each SJFT bout using the 15-point Borg’ scale [34]. The subjects were asked to report RPE using cues derived from all sensations experienced during the exercise. Twenty-five microliters (μL) of arterialized blood sample was collected from the earlobe before warm-up (rest) and immediately before and after each SJFT bout. Blood samples were immediately placed in microtubes containing 1% of sodium fluoride and centrifuged at 3000 rpm for 10-min to ensure plasma separation. Plasma lactate concentration [La] was determined with commercial kits (Biotecnica, Varginha, Brazil) using a spectrophotometer. Heart rate was measured immediately before, immediately after, and 1 min after each SJFT bout using a cardiofrequencimeter (Polar S810i heart rate monitor, Polar Electro OY, Kempele, Finland).

### 2.8. Statistical Analysis

The Kolmogorov-Smirnov test was applied to determine if the data met parametric assumptions (normality). The data were tested for homogeneity using Levene’s test. After confirmation of normality and homogeneity, the test-retest reliability of primary measurements was examined using Student’s *t*-test for paired samples, intraclass correlation coefficient (ICC) and technical error of measurement (TEM) [27]. Body weight and body fat were compared before and after weight loss using a paired *t*-test. A two-way analysis of variance with repeated measures (time × condition) was used to determine if body weight and body fat recovery post weigh-in differed between CAF and PLA. Similarly, a two-way analysis of variance with repeated measures (time × condition) followed by a Bonferroni adjustment was used to investigate if RPE, HR, [La], or performance in SJFT differed between CAF, PLA, and control conditions. When assumptions of sphericity were violated, the critical value of *F* was adjusted using the Greenhouse-Geisser epsilon value from the Mauchley test of sphericity. Total energy and percent intake of carbohydrate, fat and protein recorded during the recovery period were compared between CAF and PLA using a paired *t*-test. Confidence intervals (95% CI), effect sizes (partial eta-squared, η_p_^2^) for the t and F ratio, and power effect were also calculated when appropriate to evaluate the magnitude of differences. η_p_^2^ values of 0.1, 0.3 and 0.5 were considered as small, moderate and large, respectively [35]. The results of descriptive statistics are reported as the mean ± SD. Significance was defined as *p* < 0.05. All analyses were performed using SPSS software (version 17.0; IBM, Chicago, IL, USA).

## 3. Results

### 3.1. Test-Retest Reliability

There were no significant differences between test and retest measurements for body weight, body fat, or number of throws (Table 1; all *p* > 0.05). All variables showed a low TEM and a moderate to high ICC.

**Table 1:** Mean ± SD, intraclass correlation coefficient (ICC), and typical error of measurement (TEM) for anthropometric measurements and special judo fitness test outcome (throws) during test and retest assessments.

Variables	Test	Retest	ICC	TEM (%)
Body weight (kg)	69.1 ± 12.2	69.3 ± 12.1	0.99 (0.99–1.00)	0.3 (0.2–0.6)
Body fat (%)	10.4 ± 12.2	12.1 ± 14.0	0.99 (0.99–1.00)	0.3 (0.2–0.5)
SJFT-1 (throws)	22.3 ± 2.5	21.8 ± 1.6	0.81 (0.15–0.95)	1.2 (0.9–2.0)
SJFT-2 (throws)	21.2 ± 2.4	21.4 ± 1.9	0.87 (0.32–0.96)	1.1 (0.8–1.9)
SJFT-3 (throws)	20.8 ± 2.0	21.4 ± 1.8	0.69 (−0.35–0.93)	1.3 (1.0–2.3)
Total Throws	64.3 ± 5.9	64.7 ± 4.6	0.88 (0.50–0.97)	2.4 (1.7–4.1)

TEM and ICC are presented as the mean and confidence interval (95% CI).

### 3.2. Rapid Body Weight-Loss

Body weight was significantly reduced in both CAF (*t*(6) = 5.82, *p* < 0.05, η_p_^2^ = 0.93, 95% CI = 1.57 –4.08) and PLA (*t*(6) = 4.76, *p* < 0.05, η_p_^2^ = 0.90, 95% CI = 1.31–4.41) after the rapid weight loss period compared to control (Table 2). Total body weight lost was similar between CAF and PLA conditions (−3.9 ± 1.6 and −4.0% ± 2.3% from control, respectively). Three hours after weigh-in, body weight increased in both CAF (*t*(6) = 5.95, *p* < 0.05, η_p_^2^ = 0.93, 95% CI = 1.24–3.12) and PLA (*t*(6) = 3.25, *p* < 0.05, η_p_^2^ = 0.82, 95% CI = 0.39–3.47), but did not return to the control values (*p* < 0.05). The magnitude of recovery was similar between CAF and PLA conditions (−3.0 ± 1.3 and −2.7% ± 2.2% from control, respectively, *p* > 0.05). Body fat did not change over the time and there was no significant difference between the conditions (*p* > 0.05, Table 2).

Body weight before starting weight-loss procedures was similar between CAF and PLA conditions (72.1 ± 12.3 *versus* 72.7 ± 11.7 kg, respectively), and did not differ from control (73.0 ± 11.8 kg); this suggests that the wash-out period was sufficient to return body weight to control levels in both experimental conditions. Only one athlete distinguished correctly which capsule had been ingested (caffeine); the others did not correctly identify which capsule they had ingested.

**Table 2:** Anthropometric data from athletes during control, post-weight loss (weigh-in), and loading period for caffeine (CAF) and placebo (PLA) treatments.

Variables	Control	Weigh-In		3-H Loading Period	
		CAF	PLA	CAF	PLA
Body weight (kg)	73.0 ± 11.8	70.1 ± 11.4 ^a^	70.1 ± 11.9 ^a^	70.8 ± 11.5 ^a,b^	71.0 ± 11.9 ^a,b^
Body fat (%)	13.4 ± 11.1	12.2 ± 12.2	13.2 ± 12.1	13.1 ± 12.0	12.3 ± 11.0

Data are reported as the mean ± SD. ^a^ Significantly lower than control; ^b^ Significantly higher than weigh-in in the same condition.

Table 3 shows total energy intake and macronutrient percentages during the first three hours of recovery in CAF and PLA conditions. Total energy intake, as well as the distribution of calories from carbohydrate, fat and protein, was similar between CAF and PLA (*p* > 0.05).

**Table 3:** Energy and macronutrients intake recorded during the first three hours of recovery for caffeine (CAF) and placebo (PLA) conditions (mean ± SD).

Variables	CAF	PLA
Total energy (kcal)	674.7 ± 123.0	774.6 ± 302.2
Carbohydrate (%)	66.7 ± 11.9	59.8 ± 8.2
Fat (%)	21.6 ± 8.4	26.7 ± 6.9
Protein (%)	12.5 ± 6.2	13.5 ± 3.6

### 3.3. Performance, RPE, Plasma Lactate, and HR

**Figure 2:**
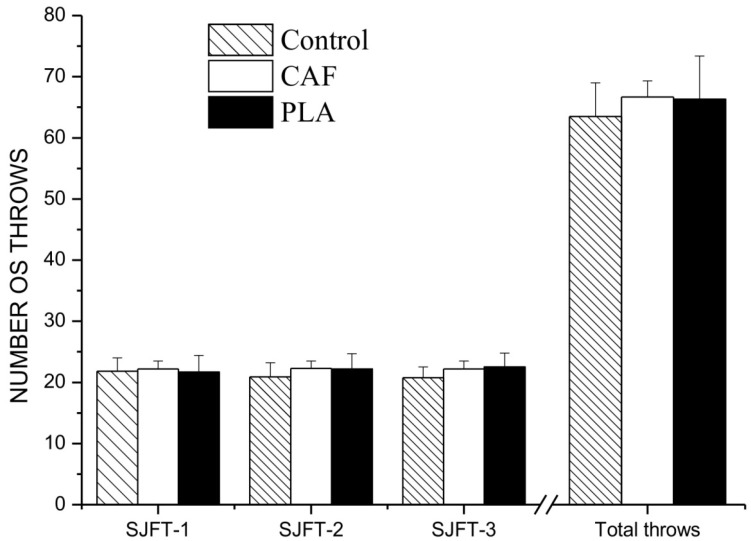
Number of throws in each bout of special judo fitness test (SJFT) in control, and after a 5-day weight-loss period followed by caffeine (CAF) or placebo (PLA). Data are expressed as the mean ± SD.

There was no significant main effect on condition (*F*(2,10) = 1.12, *p* > 0.05, η_p_^2^ = 0.18, power effect = 0.19) or bout (*F*(2,10) = 0.40, *p* > 0.05, η_p_^2^ = 0.01, power effect = 0.05) for the number of throws (Figure 2). There was also no interaction effect (*F* (4,20) = 1.07, *p* > 0.05, η_p_^2^ = 0.17, power effect = 0.27). Similarly, there was no main effect on condition (*F*(2,10) = 1.16, *p*>0.05, η_p_^2^ = 0.25, power effect = 0.27), bout (*F*(2,10) = 0.98, *p* > 0.05, η_p_^2^ = 0.16, power effect = 0.17) or interaction (*F*(4,20) = 2.47, *p* > 0.05, η_p_^2^ = 0.33, power effect = 0.59) on SJFT performance index (Table 4).

**Table 4:** Performance index during the SJFT for caffeine (CAF), placebo (PLA) and control (CON) conditions (Mean ± SD).

Condition	SJFT-1	SJFT-2	SJFT-3
CON	14.2 ± 1.9	15.8 ± 2.5	16.3 ± 2.3
CAF	14.9 ± 1.4	15.1 ± 1.3	15.3 ± 1.9
PLA	15.1 ± 2.2	14.5 ± 1.3	15.1 ± 1.5

**Figure 3:**
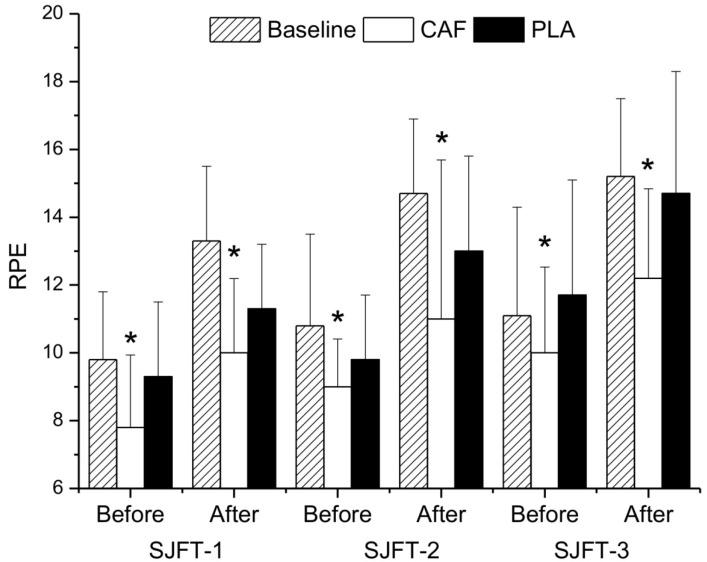
Rating of perceived exertion (RPE) before and after each bout of SJFT in control and after a 5-day weight loss period followed by caffeine (CAF) or placebo (PLA). Data are expressed as the mean ± SD. * Significantly lower than control and PLA conditions.

**Figure 4:**
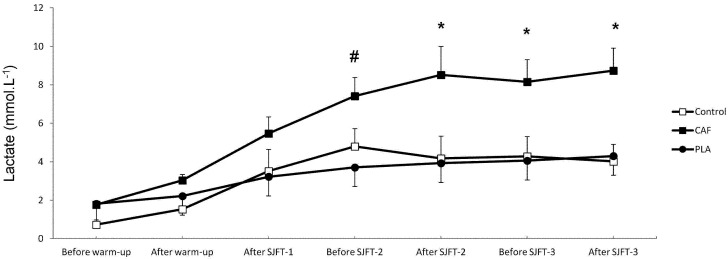
Plasma lactate concentration [La] before and after warm-up, and before and after each bout of SJFT in control and weight loss conditions followed by caffeine (CAF) or placebo (PLA). Data are presented as the mean ± SD. ^#^ CAF significantly higher than PLA at the same time point; * CAF significantly higher than control and PLA at the same time point.

However, there was a main effect of time on RPE; values increased significantly throughout SJFT in all conditions (Figure 3, *p* < 0.05). There was also a main effect of condition (*p* < 0.05), as values were lower in CAF than control or PLA. However, there was no interaction effect (*p* > 0.05). Similarly, [La] increased significantly with time in all conditions (Figure 4, *p* < 0.05). There was also a main effect of condition and an interaction between condition and time; [La] values became elevated in CAF over control or PLA as the SJFT progressed (*p* < 0.05).

HR increased significantly over time in all conditions (*p* < 0.05), but there was no effect of condition or interaction (*p* > 0.05). HR before SJFT-1 (control: 103 ± 23; CAF: 116 ± 23 and PLA: 113 ± 24 bpm) and after SJFT-3 (control: 182 ± 10; CAF: 187 ± 14 and PLA: 184 ± 14 bpm) was similar between the conditions (*p* > 0.05).

## 4. Discussion

The main findings of the present study were that caffeine, consumed after a ~5% reduction in body weight and a short loading period, did not increase performance during a high-intensity, intermittent judo-specific test. However, caffeine ingestion reduced perceived effort and increased plasma lactate responses during the performance test.

### 4.1. Body Weight Loss and Performance

In the present study, we found a significant reduction in initial body weight (~4%) after a short weight-loss period in both CAF and PLA conditions. The weight loss achieved in this study was similar to that reported by others using the same duration and with athletes of similar training status [11,13,32,33]. For example, Artioli *et al.* [32] observed a ~4% reduction in body weight after a 5-day weight loss period when compared to control values; more recently, Mendes *et al.* [33] showed a ~5% reduction in body weight after a 5-day weight loss period. Therefore, we argue that the reduction in the body weight observed in the present study was successful and consistent with values reported in the literature.

Our data also demonstrated that a ~4% reduction in body weight did not negatively affect performance during three judo-specific tests. Rapid weight loss is known to impair performance in wrestlers [10] and judo athletes [11,13] when the athletes are not allowed to re-fed and rehydrate after the weigh-in. However, weigh-in is followed by a short recovery period (~4 h) in judo competitions in which the athletes are permitted to consume foods and liquids freely [32,33,36]. Therefore, we intended to reproduce the same procedures adopted in actual competitions. Our findings confirm previous studies showing that a “loading period” after rapid weight loss rescues performance [32,33]. Importantly, total energy intake, as well as distribution of calories from carbohydrate, fat and protein, was similar between CAF and PLA during the 4-h recovery. This suggests that the diet strategy adopted during the recovery period was successfully replicated across conditions. Supporting this successful recovery strategy, body weight recovery was similar between these two conditions.

### 4.2. Caffeine and Performance

Our results demonstrated that caffeine ingestion does not improve performance in SJFT when performed after rapid weight loss. Caffeine’s performance benefits have been described for continuous exercise [23,37,38], as well as for sprints and repeated bouts of intense exercise [17,18,20]. However, it is important to note that not all studies found a positive effect of caffeine [39,40]. For example, Greer *et al.* [39] and Crowe *et al.* [40] showed that caffeine ingestion (6 mg·kg^−1^) did not have any effect on performance (peak power, average power or decrement in total work) during successive maximal cycling bouts (four bouts of 30 s or two bouts of 60 s) compared to placebo. The reasons for these differences are not clear, but may be related to different performance protocols with different durations, intensities, and types of exercise. In the present study, we used a short, high-intensity intermittent exercise specific to judo performance. We found that the number of throws (performance) during three bouts consisting of three series (1 × 15 s, and 2 × 30 s separated by 10-s recovery) with 5-min recovery periods did not differ between PLA and CAF. Data from the present study therefore suggests that caffeine ingestion does not confer additional performance benefits during a judo test when consumed after a rapid weight loss period. Part of the discrepancy with results reported in the literature may be attributed to different types of movement and skills used during SJFT. Technique is an important determinant in SJFT [14,15,16]. Unfortunately, there are no studies in the literature verifying the effects of caffeine on performance during SJFT for comparison. Additionally, in the present study SJFT was evaluated after a weight-loss period, which may have interfered with caffeine effects. Further studies should investigate the effects of caffeine on performance during the SJFT in a fresh, normal weight condition.

### 4.3. Caffeine and RPE

Caffeine ingestion reduced RPE by ~14.6% during SJFT. This result is consistent with other studies showing a reduction of RPE after caffeine ingestion [17,18,26]. This reduction could be caused by caffeine antagonism on adenosine A2 receptors in the CNS [23]. Caffeine easily crosses the blood-brain barrier by simple diffusion and competes with adenosine for adenosine A2 receptors in the CNS [41]. Adenosine enhances pain perception and induces sleepiness. Thus, caffeine can counter regulate these negative effects, reducing pain and/or effort perception [42]. It is interesting to note that Doherty and Smith [26] showed that caffeine ingestion reduced perceived exertion; 29% of the variance explaining the ergogenic effect of caffeine on performance was obtained by the decreased RPE response. However, in the present study, caffeine lowered RPE but did not improve performance. This suggests that athletes had reduced feelings of fatigue after caffeine intake, but this was insufficient to improve performance after rapid weight loss. It is possible that this reduction in RPE after caffeine ingestion may have improved performance if SJFT bouts had progressed. Further studies should investigate the effects of caffeine on performance during a match simulation (e.g., 5-min judo combat) or large SJFT number (>3).

### 4.4. Caffeine and Lactate Concentration

The caffeine ingestion increased lactate concentration from the beginning of SJFT-2 until the end of SJFT-3. Although there is no previous data investigating the effects of caffeine on plasma lactate after successive SJFT tests, some studies using cycling exercise are supportive of our findings [20,40,43]. Collomp *et al.* [20] showed that caffeine ingestion (5 mg·kg^−1^) increased blood lactate concentration after a bout of the Wingate test compared to placebo. Additionally, Crowe *et al.* [40] showed an increase in blood lactate concentration during successive cycling bouts (two bouts of 60 s each) after caffeine ingestion (6 mg·kg^−1^). Two mechanisms have been proposed to explain this increase in blood lactate. In the first, caffeine increases anaerobic activity through its antagonistic action on peripheral adenosine receptors, which could prevent the inhibitory effects of adenosine on phosphofructokinase activity in skeletal muscle [22]. Secondly, caffeine promotes catecholamine release to facilitate the conversion of phosphorylase *b* to its more active *a* form, accelerating muscle glycogenolysis [20]. However, although lactic anaerobic metabolism is fundamental during judo combats, some new findings have suggested that aerobic and lactic metabolisms are equally important [44]. Thus, even a caffeine-stimulated increase in lactic metabolism may not be sufficient to increase performance.

### 4.5. Limitations

It is important to acknowledge the limitations of the present study. Caution is advised when applying these data to internationally competitive athletes because the present study only evaluated athletes of national and regional levels. Although we did not observe impaired performance during the SJFT, a larger weight reduction (e.g., ≥10% of body mass), which can be found in an actual competition, may result in considerable performance impairment even after a loading period. However, requesting athletes to reduce more than 5% of body mass would considerably affect participant’s compliance and raise ethical concerns, thus compromising the feasibility of the study. However, judo athletes typically lose less than 5% of their body mass to compete, demonstrating that our results apply to the majority of cases in real settings [32]. Finally, we used a small sample size. Although we estimated that six participants would be sufficient, we found a reduced power effect. A larger number of participants may be considered unrealistic for studies with invasive procedures, multiple measurements, or many test days. In addition, weight-loss procedures are strenuous and difficult to follow up. This has likely contributed to similarly reduced sample sizes in a series of other studies [32,33]. However, we found a small effect size for all main performance outcomes, suggesting that any effect of caffeine was minimal and would require a non-realistic sample of more than 100 participants.

## 5. Conclusions

Caffeine ingestion, consumed during a loading period following a 5-day weight loss period, does not increase performance, but decreases RPE and increases plasma lactate concentration. This suggests that caffeine is able to reduce feelings of fatigue and increase lactic anaerobic metabolism, but without altering performance.
